# A Pressure Sensor Based on the Interaction between a Hard Magnet Magnetorheological Elastomer and a Hall Effect Structure

**DOI:** 10.3390/mi15101221

**Published:** 2024-09-30

**Authors:** Onejae Sul, Sung Joong Choo, In-Sik Jee, Jeengi Kim, Hyeong-Jun Kim

**Affiliations:** CK Materials Lab Co., Ltd., Ansan-si 15657, Gyeonggi-do, Republic of Korea; oj.sul@ckmaterialslab.com (O.S.); sj.choo@ckmaterialslab.com (S.J.C.); is.jee@ckmaterialslab.com (I.-S.J.); jg.kim@ckmaterialslab.com (J.K.)

**Keywords:** magnetorheological elastomer, Hall effect, silicon, sensor, pressure, semiconductor

## Abstract

In this article, we report a novel pressure sensing method based on the Hall effect and a hard magnet magnetorheological elastomer (hmMRE). The elastic property of the MRE under pressure was used to generate spatial variation in the magnetic flux density around the MRE, and the variation was detected by the Hall effect device underneath. As the first development in this kind of pressure sensing mechanism, we conducted research for the following three purposes: (1) to verify the Hall effect on the output signal, (2) to understand the sensor output variations under different modes of operation, and (3) to utilize the mechanism as a pressure sensor. We characterized the sensor with its operation parameters, such as signal polarity switching depending on wiring directions, signal amplitude, and offset shift depending on the input voltage. Based on the analyses, we concluded that the Hall voltage represents the pressure applied on the hmMRE, and the new pressure sensing mechanism was devised successfully.

## 1. Introduction

The most dominant mode of pressure sensing in industrial applications is piezoresistivity. Typically, a doped silicon wafer is etched through its thickness to form a thin flexible membrane. Then, a Wheatstone bridge circuit is fabricated on the membrane to detect the flexure of the membrane depending on the pressure. This kind of design is very popular because of its simple operational principle, performance uniformity, and reliability. However, the design involves a deep etching process, which needs a separate substrate requiring wire bonding with a driving circuit, i.e., an application-specific integrated circuit (ASIC). Also, typically, a milliamp current is required to obtain the sensor output, which cannot be ignored for energy-hungry applications, such as battery-powered remote operation or wearable operation.

In search of another pressure sensing mode that enables simpler fabrication without deep etching and power reduction, we devised a new one using magnetism. In this sensing mode, a Hall effect device is used to detect magnetic flux density variation, and the magnetic field variation is supplied by an elastic permanent magnetic material, i.e., a hard magnetic magnetorheological elastomer (hmMRE). Because our hmMRE contains micro NdFeB particles, it has magnetization without an external field after the film undergoes a magnetization process. The hmMRE is a film located above the Hall effect device with a cavity. Then, pressure impinging on the top surface of the hmMRE film produces a flexure, and the strength of the magnetic flux density generated by the film varies at the device. If calibrated properly, the Hall voltage can represent the amplitude of the pressure applied on the hmMRE.

Conventional MREs, which contain soft magnetic material like CIPs (carbonyl iron particles), have been used for pressure, haptic, and tactile sensing. In an earlier MRE sensing research study, the resistivity of an MRE itself was used for sensing. CIP material was mixed with rubber [1], and it showed conductance variation depending on magnetic flux density and pressure. A similar result with CIP material in silicone rubber [2,3] was reported as piezoresistivity depending on mechanical stimuli. Later, in 2018, Kawasetsu et al. [4] reported a sensing scheme based on the spatial variation in magnetic flux density around an MRE film. They used planar coils and a permanent magnet underneath to pick the field disturbances around the coils and detect the pressure on the MRE. Conductivity-enhanced MRE was also reported by Shabdin et al. [5]. They mixed graphite particles in an MRE, and the elastomer showed a change in resistivity and moduli depending on the magnetic flux density and force. Another study reported a haptic sensor based on an MRE and Hall effect sensors [6]. Permanent magnets were located below the sensors, and any field disturbances induced by MRE deformation could be detected by the sensors. Usually, the term MRE refers to an elastomer mixed with CIPs and natural or synthetic rubber, but there are also magnetic elastomers made up with different ingredients. Mieta et al. [7] reported a magnetoelastic material based on silver-coated magnetite particles in polydimethyl siloxane (PDMS). For more information, readers can refer to a review article in the field of MRE research [8] and to an article about an important issue, the effect of the shape and size of filler particles on the aggregation and sedimentation in MRE [9].

A magnetorheological plastomer (MRP) was also reported. The MRP is similar to an MRE in terms of the fabrication process, but it is made with a stiffer, plastic-like matrix, such as thermoplastic or thermosetting polymers. But still, under magnetic flux density, the magnetic particle can form a chain structure. Xu et al. [10] used this phenomenon to develop a dual sensing-mode, magnetic flux density, and pressure sensing device. Under magnetic flux density, because of particle chaining, more ions at the anode and cathode electrodes could pass through channels between the chains and generate higher potential between the electrodes. They observed a potential dependence variation by pressure and magnetic flux density. Amlee et al. [11] also developed a hydrogel-based MRP mixed with graphite.

Other than pressure, haptic, and tactile sensing, MREs were also used for sensing difference physical quantities, like magnetic flux density [12,13], resistivity [14], and strain [15,16]. For information on the sensor applications of MREs, readers can refer to [17,18].

In this article, we fabricate a non-traditional MRE, i.e., an hmMRE, and use it to characterize sensor responses to variations in electrical wirings, output offset by the Hall effect, and modulation of output by the input voltages. We prove experimentally that the detection of magnetic flux density variation by hmMRE deformation on a Hall effect device can be a feasible method for detecting pressure. Although our sensing mechanism is similar to Im et al.’s work, our sensing mechanism is different because it lacks permanents magnets. In addition, our sensor is a microelectromechanical system (MEMS)-based pressure sensing device, and theirs is a haptic sensing system. Finally, we discuss the power consumption reduction compared to commercial sensors.

## 2. Operation Principle and Fabrication

**Figure 1:**
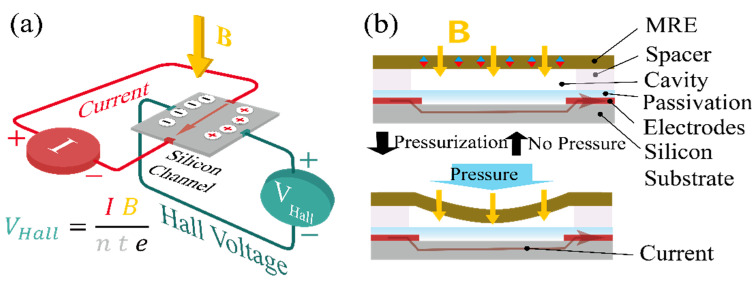
(**a**) The Hall effect. (**b**) Cross-sectional schematics of the working mechanism of pressure sensing (top) before and (bottom) after pressure application.

Figure 1 illustrates a pressure sensor system based on a Hall effect structure and an hmMRE. The Hall effect refers to a phenomenon where a moving electron under magnetic flux density (B) experiences the Lorentz force, $\vec{F}$ which has direction and magnitude defined by

$$\vec{F}=-q\left(\vec{v}\times \vec{B}\right)$$

where *q* refers to positive unit charge, $\vec{v}$ refers to the drift velocity vector of the electron, and $\vec{B}$ refers to the vector of the flux density. The hmMRE is positioned on top of the Hall effect structure, which consists of a silicon channel through which a current is applied. When no pressure is applied to the hmMRE (top diagram on the right), the magnetic flux density generated by the hmMRE and passing through the Hall sensor remains undisturbed, leading to a relatively constant Hall voltage (*V_Hall_*), as defined by the equation *V_Hall_* = *IB*/*nte*. In this equation, *I* represents the current through the silicon channel, *n* is the carrier density, *t* is the thickness of the silicon channel, *e* is the elementary charge, and *B* is the magnetic flux density.

However, when pressure is applied to the top surface of the hmMRE (bottom diagram on the right), the hmMRE deforms, causing spatial variation in the magnetic flux density distribution across the Hall sensor. This deformation results in a change in the local magnetic flux density experienced by the silicon channel and, consequently, in the Hall voltage, *V_Hall_*, in response to the applied pressure. The relationship between the applied force and the resulting Hall voltage enables the system to quantify the pressure applied to the hmMRE surface. Thus, the system effectively functions as a pressure sensor, translating mechanical forces into electrical signals through the Hall effect influenced by the deformation of the hmMRE.

**Figure 2:**
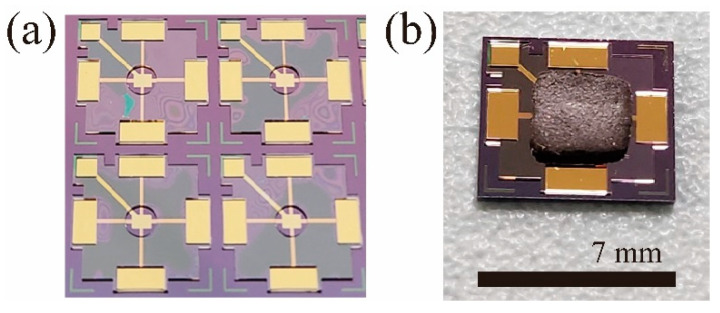
(**a**) Optical microscope image of an array of Hall devices on a wafer before dicing. (**b**) An optical image of a sensor with an MRE mounted on the Hall device.

The Hall effect-based pressure sensor was fabricated through MEMS processes. The process begins with a lightly p-doped Si substrate and a thin layer of oxide on top of it. An n-type silicon channel with a rectangular form, 200 μm in length and 100 μm in width, was created using a standard ion implantation process. Following this, a passivation oxide layer was deposited, and electrical contact areas were selectively etched. Metal electrodes were then deposited at the contact areas. After completing the metallization process, the structure was encapsulated with a second passivation layer. An electric field shielding metal layer was deposited on top of the passivation layer to reduce electric field-induced noise. After the third passivation layer, a spacer structure was fabricated to define the area for the hmMRE deflection and its mount. Finally, the hmMRE was mounted on top of the Hall sensor, completing the device structure, using a plasma-assisted bonding process [19,20]. Figure 2a shows an optical microscope image of the fabricated Hall devices. Figure 2b presents an image of a completed sensor with an hmMRE mounted on top.

The fabrication steps of our MRE are as follows. First, the liquid silicone base (Sorta-Clear from Smooth-On Inc., Macungie, PA, USA, grade A12) and crosslinker were mixed at a 1:1 ratio. As-purchased NdFeB powder (Meiqi Industry & Trade Co., Gongyi, Zhengzhou, China, grade YMM-15-7) was milled using a planetary milling machine with 3 mm Zr balls in stainless steel bowls. The NdFeB flake size after the milling process was around a few tens of micrometers. Then, the milled powder was added, the mixed paste was poured on a glass substrate, and an applicator was used to obtain a uniform film thickness of around 700 μm. The thermal curing was performed in an oven. After curing, the film was separated from the glass substrate, and it was cut into pieces a few centimeters in size. Finally, the films were magnetized by a high-power electromagnet (model STM-2520 from Magnetizing Total Solution, Dongducheon, Gyeonggi-do, Republic of Korea). The films were cut similarly to the MRE in a size of 3 mm by 3 mm, and their magnetic flux densities at their surface were measured using a gauss meter (TM-801EXP from Kanetec, Ueda, Nagano, Japan). Typically, the strength of the magnetic flux density is in the range of 300~600 gauss. We chose one near 420 gauss for our sensor.

**Figure 3:**
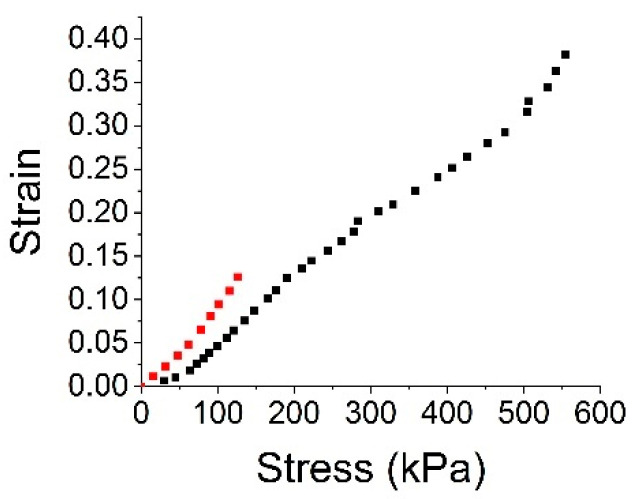
Stress–strain measurements from the thick (red) and thin (black) hmMRE specimens.

For mechanical property investigation, stress–strain curves on the fabricated hmMRE were obtained by tensional tests using a push–pull gauge (EMX-1000N II from Imada, Toyohashi, Aichi, Japan). We prepared two specimens of the hmMRE, where the first one had dimensions of 90 mm × 20 mm × 0.4 mm (thin specimen) and the second one had dimensions of 70 mm × 18 mm × 1.8 mm (thick specimen). Figure 3 shows the calculated stress–strain curves of both specimens from their force–compression measurements. Their magnetization values, i.e., the magnetic flux densities on their surface at the midpoint in their length direction, were 34 ± 5 gauss and 290 ± 50 gauss. The standard deviations come from the fluctuation in the measurement value depending on exact measurement locations, and the values do not show any significantly noticeable dependency on the elongation.

**Figure 4:**
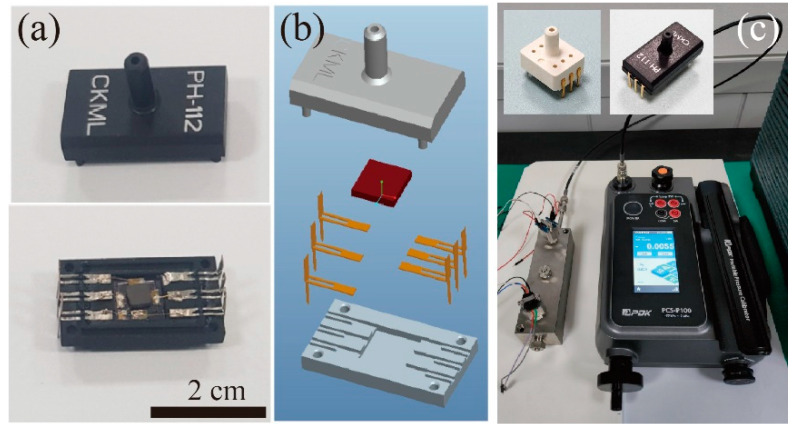
(**a**) Pictures of the assembled sensor inside cases. (**b**) Schematic design of cases and lead frames. (**c**) Pressure application and measurement setup, reference sensor (left inset), and Hall effect pressure sensor (right inset).

Figure 4a presents the images of the top and bottom cases used to package the Hall effect pressure sensor. The top image shows a central protruding nipple that acts as the inlet for applying pressure. The bottom image provides a view of the interior, showing the pressure sensor placed at the center of the bottom case. The electrical pads at the edges of the sensor are electrically connected to the lead frames of the bottom case through a wire bonding process with gold wires.

Figure 4b displays the design schematics of the packaging structure. The zoomed view includes the top case, lead frames, pressure sensor, and bottom case. The central element, colored in red, represents the Hall effect sensor. The lead frames are positioned around the periphery, designed to interface with the sensor’s pads and provide electrical connections that extend to the outside of the package.

Figure 4c illustrates the experimental setup used for applying and measuring pressure on the sensor. The setup includes a precision pressure applicator (model PCS-P100 by pressure development of Korea, PDK, Daejeon, Republic of Korea) connected to a three-port pressurizing jig via a tubing system. The applicator allows for the manual application of pressure up to 20 bar (=2000 kPa) using a lever to the jig through the tubing. The inlets of the sensors were plugged into the pressure output holes in the jig and the sensors were locked by bolts. To measure the pressure numerically, a commercial reference pressure sensor was installed in the jig together. When the pressure applicator applied pressure, the reference sensor and our sensor received an identical magnitude of pressure. An operating voltage was supplied to each sensor using two power supplies. The response voltages of the two sensors were recorded simultaneously using two-channel source meters (model 2602A by Keithley, Beaverton, OR, USA).

## 3. Results

### 3.1. Sensor Calibration

**Figure 5:**
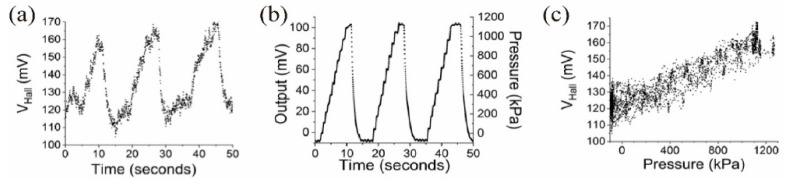
(**a**) Sensor response when the supply voltage was 10 volts. (**b**) Response of the reference sensor when the supply voltage was 5 volts, measured simultaneously with the Hall effect pressure sensor in (**a**). (**c**) Calibrated pressure response of the Hall pressure sensor.

Figure 5a,b present the measurement results of the Hall voltage (*V_Hall_*) from the developed Hall effect pressure sensor and that of the reference sensor as a function of time. The two sets of measurements were obtained simultaneously during three cycles of pressure application and relaxation. Figure 5a demonstrates a clear, repeatable response from the applied pressure over the multiple cycles. *V_Hall_* varies between approximately 100 mV and 170 mV, reflecting the dynamic changes in the applied pressure during the measurement period.

Figure 5b shows the measurement results from the reference pressure sensor with the pressure calibration results. According to the specification of the sensor, its full-scale output is 90 mV at 980 kPa. The output of the reference sensor is plotted against both time and pressure, revealing a direct correlation between the sensor’s output and the applied pressure, agreeing with the specification. The pressure applied during this test ranged from 0 kPa to approximately 1200 kPa (12 Bar). The step-like features originate from the manual lever action of the pressurizing instrument. Because the pressure applied on our sensor and the reference sensor is identical at each moment, we can plot Figure 5c, combining the response of our sensor against the pressure information at each temporal moment.

Figure 5c illustrates the resulting calibration curve of our Hall effect pressure sensor. *V_Hall_* from our sensor is plotted as a function of the applied pressure. The data reveal a correlation between the applied pressure and the Hall voltage, confirming the sensor’s capability to transduce pressure into measurable electrical signals. As the pressure increases from 0 to 1200 kPa, *V_Hall_* increases linearly from approximately 100 mV to 170 mV. This linear relationship is essential for the sensor’s application in precise pressure measurements, as it allows for the direct conversion of the Hall voltage into pressure values. There are multiple dense data points along the pressure axis because of the momentary pause in the pressure increase while the lever returns after a pressure application stroke.

The calibration results demonstrate that the developed Hall effect pressure sensor is not only responsive to pressure variations but also provides a linear output that is directly comparable to a calibrated reference sensor, validating the performance and accuracy of the sensor for potential use in various pressure sensing applications.

One important point to note is the power consumption. While the reference sensor consumes about 10 mW (5 V × 2 mA), our sensor consumes only about 1.8 mW (10 V × 18 μA), which is less than 20% of the reference sensor. The typical power consumption of commercial pressure sensors based on piezoresistivity ranges from 1 to 10 mW. Our measurement suggests that the new pressure sensor based on magnetism can be advantageous in battery-powered applications.

### 3.2. Sensor Responses Depending on Wiring and Measurement Directions

**Figure 6:**
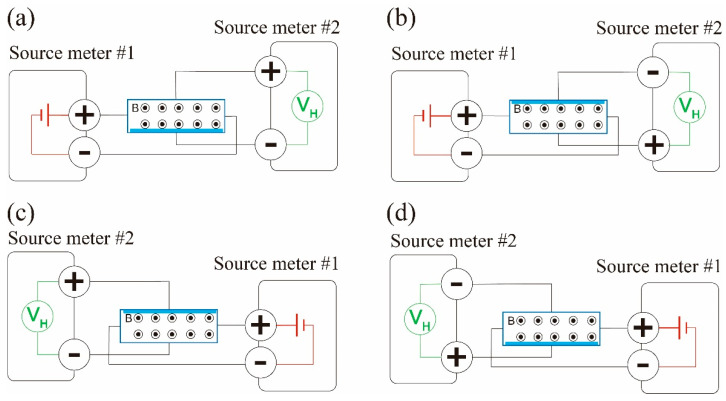
(**a**) Positive forward (P-F); (**b**) positive reverse (P-R); (**c**) negative forward (N-F); and (**d**) negative reverse (N-R) configurations. The blue area indicates the electron accumulation location.

Because the shape of the channel of the Hall device has two symmetrical axes perpendicular (one axis along the left and right ends of the rectangular channel, the other one along the top and bottom ends of the channel), one might be interested in the change in the response (*V_Hall_*) depending on the direction of current and on the measurement directions. Because of the direction of the magnetic flux density and the direction of the Lorentz force applied on the electrons, switching any pair of electrodes will reverse *V_Hall_*. Thus, we measured the responses of the sensor using four wiring configurations, as depicted in Figure 6 below. The figure shows positive forward (P-F) in (a), positive reverse (P-R) in (b), negative forward (N-F) in (c), and negative reverse (N-R) in (d). In the positive configurations, current flows from the left electrode to the right one of the silicon channel, while in the negative configurations, current flows from the right electrode to the left one. In the forward configurations, the Hall voltage is measured with the top electrode as the positive terminal and the bottom one as the negative terminal of the measurement instrument (they are called Hall probes together), whereas in the reverse configurations, the polarity of these terminals is inverted. Note that the electrode pairs, the left and right electrodes, were connected to a source meter, while the top and bottom electrodes were connected to another source meter, and the negative terminals of each source meter were not at the same potential.

**Figure 7:**
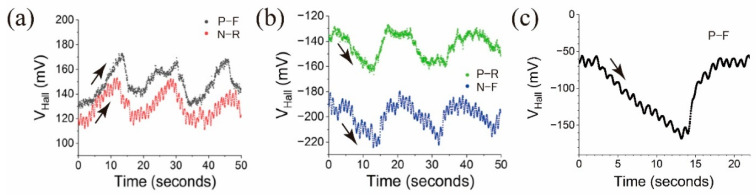
(**a**) Pressure responses in the P-F and N-R configurations; (**b**) pressure responses in the P-R and N-F configurations; and (**c**) pressure response in P-F when the north pole of the MRE faces the channel. The arrows in the figures signify the response direction of the sensor from initial *V_Hall_* (offset) value.

Figure 7 shows the results of experiments conducted to analyze the behavior of the Hall effect pressure sensor under different measurement configurations. Figure 7a,b depict *V_Hall_* as a function of time under several pressure cycles for the four different measurement configurations. The arrows in the graphs show the direction of *V_Hall_* change by the pressure deviating from the starting levels (offsets).

The data in Figure 7a show clear responses of the sensor under three consecutive pressure cycles across the Hall probes. The initial positive offset can be understood by the Lorentz force. When the south pole of the hmMRE faces the silicon channel (field directing out of the page, as in Figure 6), the electrons will experience force toward the bottom Hall probe. Thus, they will generate negative potential at the bottom electrode. If a force is applied to the hmMRE, then the strength of the magnetic flux density increases, and more electrons are accumulated near the bottom probe, which results in the increase in the Hall voltage, as seen in Figure 7a.

When any pair of electrodes were switched, the sensor responses were reflected against zero potential. In Figure 7b, in the normal reverse (N-R) and switched forward (S-F) configurations, the potential difference between the Hall probes is mirrored against the data in Figure 7a.

Figure 7c demonstrates the effect of changing the magnetic polarity of the hmMRE on the sensor’s output. Under the same positive forward (P-F) measurement configuration using another sensor, the Hall voltage signal was observed to reverse its polarity when the magnetic flux density at the channel was reversed, such that the north pole of the hmMRE faced the silicon channel instead of the south pole. This reversal in signal polarity is another direct consequence of the reversed field direction applied to the Hall sensor, which causes a corresponding change in the sign of the Hall voltage, consistent with the Hall effect’s dependence on the direction of the applied magnetic flux density.

The results demonstrated in Figure 7 give direct evidence of the Hall effect on what we observed from the Hall probe measurements. They also demonstrate the sensitivity of the developed Hall effect pressure sensor to both the electrical measurement configuration and the magnetic orientation of the hmMRE.

Another interesting aspect of our sensor is the variation in *V_Hall_* as a function of the hmMRE flux density. We fabricated three sensors using hmMREs with different flux densities. Using the hmMREs with 384, 420, and 580 gauss each, the sensors showed *V_Hall_* of 22, 42, and 79 mV at an identical supply voltage of 10 V under 800 kPa pressure. This result shows an increasing trend in *V_Hall_* following the increasing flux density; the number of fabricated sensors is insufficient to access the dependency statistically. This topic is our future research direction.

### 3.3. Offset Regulation by Grounding

**Figure 8:**
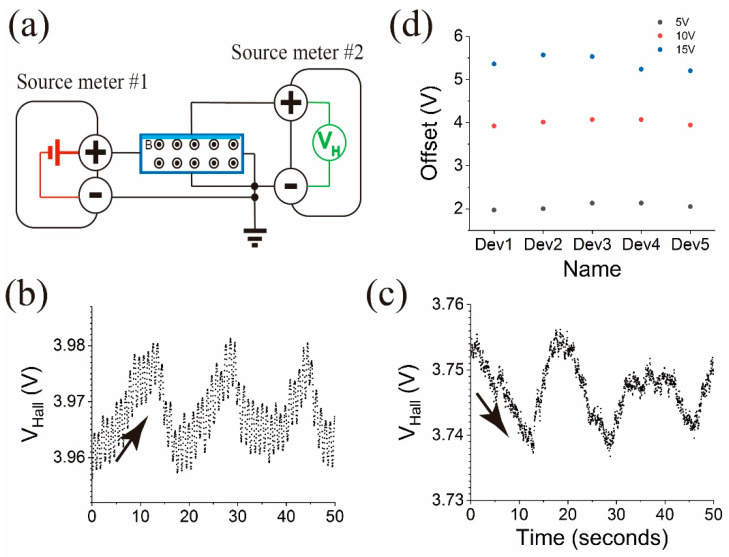
(**a**) Revised measurement scheme with ground connection. (**b**) The sensor response in the P-F configuration and (**c**) the response in the P-R configuration. The arrows in (**b**) and (**c**) signify the response direction of the sensor from initial *V_Hall_* (offset) value. (**d**) Dependency of the supply voltage of the offset locations measured in five different sensors.

We fabricated a few tens of sensors, and we observed the same mirrored responses when switching the wirings. The offsets, similarly observed in Figure 7, were different from sensor to sensor, and they were not predictable. We chose to set the negative terminals of the source meters to electrical ground to remove the randomness of the offset locations. In the next measurements, we connected the negative terminals of the two source meters to the ground terminals of each instrument, and the two ground terminals were connected together. Figure 8a shows the wiring configuration and Figure 8b,c show the sensor responses under this configuration change.

Figure 8 illustrates the effect of grouping the two negative terminals together and connecting them to the ground on *V_Hall_* and the resulting responses of the same sensor in Figure 7. This change in grounding configuration altered the baseline offset location near 3.96 V in the P-F configuration (Figure 8a,b) and 3.75 V in the P-R configuration (Figure 8c while maintaining the sensor’s ability to measure pressure variations. The offset difference between the P-F and P-R configurations comes from the Hall effect by the hmMRE. When the hmMRE was removed from the identical sensor, the offset difference was reduced from 180 mV to 35 mV, implying that the major reason for the offset difference is the Hall effect. The 35 mV difference is believed to come from the internal circuitries of the measurement instrument.

Figure 8d displays the offset voltage for five different devices (Dev1 to Dev5) across three different supply voltages (5 V, 10 V, and 15 V). The data reveal a clear relationship between the supply voltage and the offset location, with higher supply voltages producing higher offset levels. This suggests that the increased input voltage also increased the potential at the top electrode. At 5 V, the offset voltage is 2.05 ± 0.07 V across all devices, while at 10 V and 15 V, the offsets are 4.00 ± 0.07 V and 5.38 ± 0.16 V, respectively.

As the final measurements, we observed sensor responses under different pressures while maintaining the grounding method.

**Figure 9:**
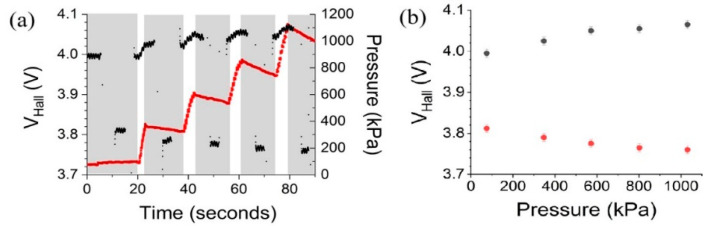
(**a**) *V_Hall_* under increasing pressure steps in two measurement configurations, P-F and N-F. (**b**) A plot based on (**a**). Black dots are *V_Hall_* measurements of P-F configuration, and red ones of N-F configuration.

Figure 9 presents the relationship between *V_Hall_* and applied pressure for the Hall effect pressure sensor under the same grounding configuration.

In Figure 9a, *V_Hall_* is plotted in black as a function of time, while the applied pressure is overlaid for comparison in red. The shaded areas indicate pause periods between the pressure pumping duration (white areas). The pressure was increased stepwise up to approximately 1000 kPa. The small pressure decrease after the stepwise increase was due to the pressure leak at the casing. The intervals where no *V_Hall_* data were recorded were due to the change in the wiring, i.e., switching between positive and negative wiring configurations. As the pressure increased, there was a corresponding stepwise increase in *V_Hall_*.

Figure 9b summarizes the response of the Hall voltage to the applied pressure. Here, the Hall voltages were recorded at the moment when the moment pressure step was generated. The voltages are plotted against pressure for two different conditions, P-F and N-F, indicated each by the black and red data points.

These results confirm that the Hall effect pressure sensor exhibits a reliable output in response to varying pressure levels, and the grounding configuration primarily affects the offset rather than the sensor’s sensitivity to pressure.

## 4. Discussion and Conclusions

In this article, we first obtained proof of the Hall effect on the response of the sensor as a new pressure-sensing device. Then, we characterized its responses under various measurement schemes. The results from the sensors demonstrate its robust and reliable performance in translating mechanical pressures into electrical signals. The calibration of the sensor revealed a consistent and repeatable response to applied pressure, showing a clear linear relationship between *V_Hall_* and the applied pressure, ranging from 0 to a maximum of 1200 kPa. This linearity is crucial for the sensor’s functionality in precise pressure measurement applications, where direct conversion from voltage output to pressure values is required.

Additionally, the experiments examined the sensor’s behavior under different wiring configurations and magnetic orientations of the hmMRE. It was observed that switching electrode configurations reversed the polarity of *V_Hall_*, which is attributed to the Lorentz force acting on the electrons. The results from these different configurations confirm the sensitivity of the Hall sensor to both the direction of the wiring and the magnetic field, providing evidence of the Hall effect’s influence on the sensor’s performance. The grounding experiments further revealed that the sensor’s baseline voltage offset was dependent on both the grounding scheme and the supply voltage.

The Hall effect pressure sensor based on hard magnet magnetorheological elastomers demonstrated a reliable ability to detect and quantify pressure with a high degree of linearity. These findings indicate that the sensor is well-suited for applications where precision and low-power consumption are critical, particularly in battery-powered or wearable technologies.

Further optimizations of the sensor’s electrical configurations could enhance its sensitivity and robustness, making it a compelling alternative to traditional commercial piezoresistive pressure sensors.

## 5. Patents

There is a pending patent with the title, “Pressure sensor using magnetic field” (Application number 10-2023-0120362 from the Korean intellectual property office).

## Data Availability

The original contributions presented in the study are included in the article, further inquiries can be directed to the corresponding authors.
